# Brap2 Regulates Temporal Control of NF-κB Localization Mediated by Inflammatory Response

**DOI:** 10.1371/journal.pone.0058911

**Published:** 2013-03-15

**Authors:** Osamu Takashima, Fuminori Tsuruta, Yu Kigoshi, Shingo Nakamura, Jaehyun Kim, Megumi C. Katoh, Tomomi Fukuda, Kenji Irie, Tomoki Chiba

**Affiliations:** 1 Graduate School of Life and Environmental Sciences, University of Tsukuba, Tsukuba, Japan; 2 Graduate School of Comprehensive Human Sciences, University of Tsukuba, Tsukuba, Japan; IISER-TVM, India

## Abstract

Nuclear factor-kappaB (NF-κB) is critical for the expression of multiple genes involved in inflammatory responses and cellular survival. NF-κB is normally sequestered in the cytoplasm through interaction with an inhibitor of NF-κB (IκB), but inflammatory stimulation induces proteasomal degradation of IκB, followed by NF-κB nuclear translocation. The degradation of IκB is mediated by a SCF (*S*kp1-*C*ullin1-*F*-box protein)-type ubiquitin ligase complex that is post-translationaly modified by a ubiquitin-like molecule Nedd8. In this study, we report that BRCA1-associated protein 2 (Brap2) is a novel Nedd8-binding protein that interacts with SCF complex, and is involved in NF-κB translocation following TNF-α stimulation. We also found a putative neddylation site in Brap2 associated with NF-κB activity. Our findings suggest that Brap2 is a novel modulator that associates with SCF complex and controls TNF-α-induced NF-κB nuclear translocation.

## Introduction

The mammalian NF-κB family plays a central role in the regulation of a wide variety of cellular functions, such as inflammatory response, cell cycle, and survival [1], [2]. The NF-κB family is composed of five members, RelA/p65, c-Rel, RelB, NF-κB1 (p50 and its precursor p105), and NF-κB2 (p52 and its precursor p100). All of the NF-κB members contain an N-terminal Rel-homology domain (RHD), which is responsible for dimerization, nuclear translocation, DNA binding, and interaction with IκB. Normally NF-κB associates with IκB in the cytoplasm. However, stimulation with inflammatory cytokine including TNF-α, IL-1β, and Toll-like receptor ligands activates the NF-κB pathway [1], [2]. This pathway conducts signals to the IκB kinase (IKK) complex, which is composed of IKKα, IKKβ, and the regulatory subunit NF-κB essential modulator (NEMO), leading to phosphorylation of IκBα [3]. This phosphorylation of IκBα is essential for its recognition by ubiquitin ligase SCF^β-TrCP^, which induces ubiquitination and degradation of IκBα in a phosphorylation-dependent manner. Finally, NF-κB translocates from the cytoplasm to the nucleus, inducing target gene expression [4], [5]. Although a substantial number of studies have reported that SCF^β-TrCP^ plays an important role in the translocation of NF-κB, it is not clear how temporal control of NF-κB translocation is coordinated.

Brap2 was initially identified as a protein that interacts with the breast cancer tumor suppressor protein, BRCA1 [6]. Brap2 functions as an E3 ubiquitin ligase through the RING-finger domain and modulates the Ras-MAPK pathway by regulation of auto-ubiquitination [7], [8]. In addition, genetic disruption of Brap2 in *C. elegans* impairs expression of p21 in response to oxidative stresses [9] and Brap2 acts as an anchor protein for p21 through direct interaction [10], suggesting that Brap2 can control different kinds of intracellular signals. Interestingly, recent genome-wide analyses have revealed that Brap2 is associated with several human disorders caused by inflammatory dysfunction, including myocardial infarction, carotid atherosclerosis and central obesity [11]–[13]. In addition, Brap2 expression is induced by inflammatory stimulation such as lipopolysaccharide (LPS) [12]. Thus it is important to reveal the function of Brap2 to treat these diseases and develop the therapeutics.

Nedd8, a ubiquitin-like (UBL) protein, covalently conjugates with the ε-amino group of lysine residue in several proteins and modulates biochemical and functional properties of target proteins. Nedd8 plays crucial roles in physiological processes such as cell cycle and signal transduction, and membrane trafficking [14], [15]. It has recently been reported that several proteins including Cullin family proteins, p53, Mdm2 and RPL11 are neddylated *in vivo* and *in vitro* [16]–[20]. Covalent conjugation of Nedd8 to a target protein seems to regulate either enzymatic activity or binding affinity against another protein. Also neddylation is thought to influence the stability of the target protein [19]–[24], indicating that modification of Nedd8 is suitable for control of protein properties. Disruption of the *ned8* gene in fission yeast causes proliferation defects, and deletion of the *uba3* gene, a component of the Nedd8 E1 enzyme in mice, results in early embryonic death in utero [25], [26]. Moreover, inhibition of the neddylation cascade using a specific inhibitor, MLN4924, causes cell cycle defects and apoptosis [27]. Therefore, the neddylation cascade is a key mechanism that governs the molecular basis of proliferation, differentiation and survival. However, despite the importance of neddylation cascade in cells, the mechanisms of how neddylation controls protein functions are not fully understood. Similar to other ubiquitin-like proteins such as SUMO, Nedd8 modification may act as a landmark recognized by other proteins, which alter neddylated protein property through interaction.

In this study, we identified Brap2 as a novel Nedd8-binding protein using yeast two-hybrid screening. Brap2 associates with SCF complexes and suppresses NF-κB translocation to the nucleus. In addition, we found that Brap2 is neddylated at lysine-432 residue associated with NF-κB activity. Taken together, our data demonstrate that Brap2 is a novel modulator that controls NF-κB translocation through its capacity to associate with the SCF ubiquitin ligase and Nedd8.

## Results

### Brap2 is a novel binding protein of Nedd8

**Figure 1:**
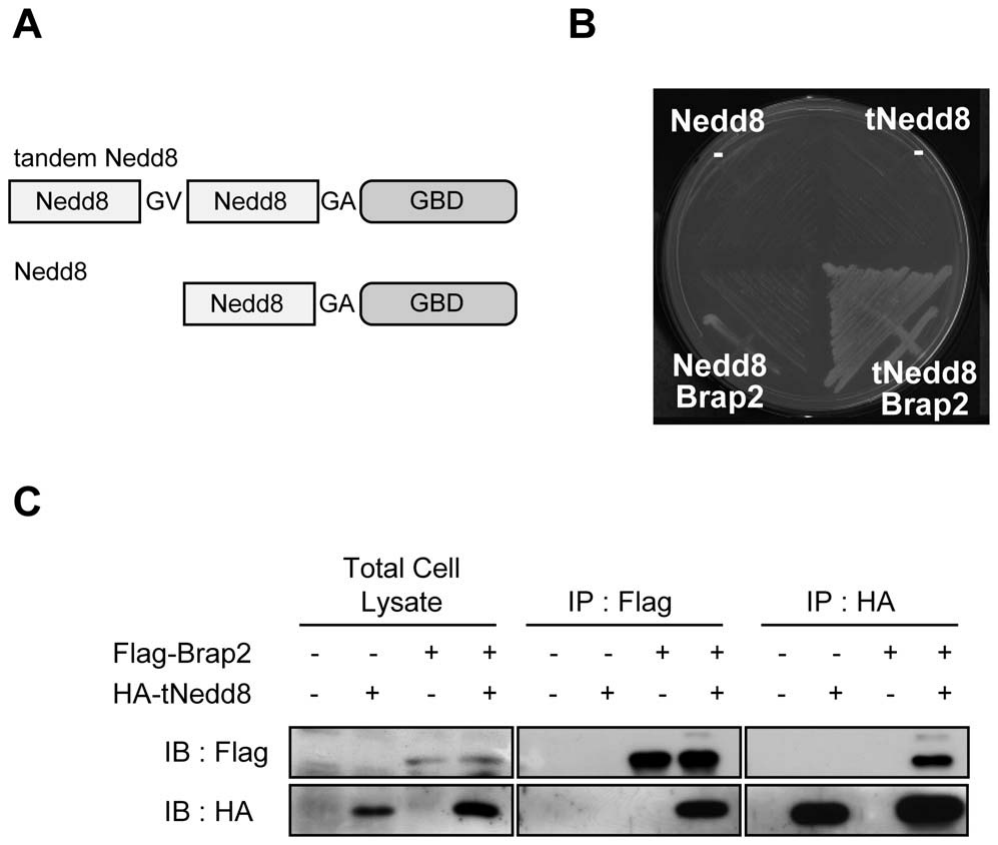
Brap2 associates with Nedd8. (A) Schematic structure of the Nedd8 constructs for yeast two-hybrid screening. **(B)** Yeast PJ69-4A strains were transformed with expression vectors as indicated. Individual transformants were streaked to synthetic medium plates lacking tryptophan, leucine, histidine. (**C**) HEK293 cells were transfected with expression vectors as indicated and were subjected to immunoprecipitation (IP) with indicated antibodies. The total cell lysates and immunoprecipitants (IP) were subjected to immunoblot (IB) analyses with antibodies to Flag and HA.

**Figure 2:**
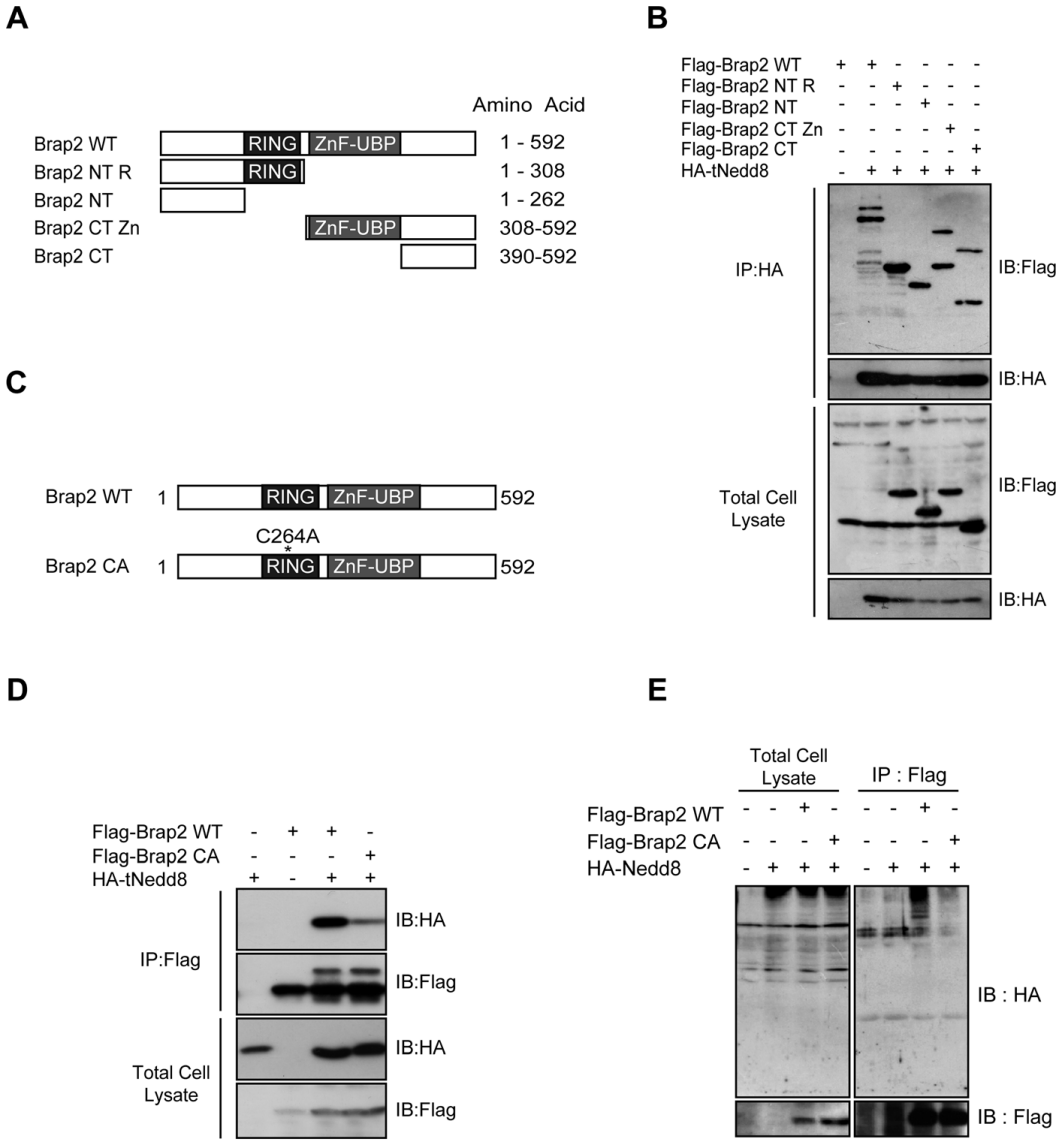
The RING domain of Brap2 is responsible for the interaction with Nedd8. (A) Schematic structure of deletion mutants of Brap2. (**B**) HEK293 cells were transfected with indicated plasmids and were subjected to immunoprecipitation (IP). The immunoprecipitants (IP) and the total cell lysates were immunoblotted (IB) with indicated antibodies. (**C**) Schematic structure of point mutant of Brap2. (**D**) HEK293 cells were transfected with expression vectors as indicated and were subjected to IP. (**E**) HEK293 cells were transfected with HA-Nedd8 and Flag-Brap2, immunoprecipitated with Flag antibody. The total cell lysates and IP were immunoblotted with HA antibody.

In an attempt to identify Nedd8-binding proteins, we performed yeast two-hybrid screening. Because tetramer formation of ubiquitin plays a pivotal role in the interaction with the proteasomal subunit Rpn10 by increasing the binding affinity [28], [29], we hypothesized that two or more copies of UBL proteins would have a strong affinity for their targets. Therefore, we made a fusion protein of GAL4 binding domain (GBD) and two copies of the Nedd8 (tandem Nedd8: tNedd8) (Fig. 1A). A single moiety of Nedd8 fused to GAL4 was also constructed as a control. The C-terminus of each Nedd8 moieties was mutated so as it would be resistant to the endogenous Nedd8 cleaving enzymes, and mimic tandem-neddylated and mono-neddylated protein. By screening mouse embryonic cDNA library, we identified Brap2 as a protein that interacts with tandem-neddylated protein but not mono-neddylated protein (Fig. 1B). The binding of Brap2 and Nedd8 in the cells were then confirmed by co-immunoprecipitation assay. We introduced Flag-Brap2 and HA-tNedd8 plasmids into HEK293 cells, and immunoprecipitated them with HA-tNedd8 using anti-HA antibody. The immunoprecipitation of HA-tNedd8 resulted in co-immunoprecipitation of Flag-Brap2, suggesting that Brap2 interacts with tNedd8 in cells (Fig. 1C). Conversely, immunoprecipitation of Flag-Brap2 caused co-immunoprecipitation of the HA-tNedd8 (Fig. 1C). Similar to ubiquitin, regular Nedd8 formed a smear band (Fig. S1) and was rarely detected as a monomeric size when Brap2 was co-expressed, at least in our systems (data not shown). Therefore it was technically difficult to determine whether monomeric Nedd8 binds to Brap2 using an immunoprecipitation assay. Instead, Brap2 could co-immunoprecipitate the neddylated smear bands suggesting that Brap2 binds to neddylated proteins that potentially include poly-neddylated and multiple mono-neddylated proteins (see Fig. 2E). Taken together, these data suggest that Brap2 associates with Nedd8 *in vivo*.

We next investigated which domain of BRAP2 is important for the binding with tNedd8. To test this, we made several deletion constructs (Fig. 2A). Unexpectedly, tNedd8 could co-immunoprecipitate all constructs examined, suggesting that BRAP2 may have multiple domains capable of binding with Nedd8 (Fig. 2B). Interestingly, the constructs that contain the CT domain (WT, CT Zn, CT) were detected as doublet bands. These bands turned out to be covalent modification of Brap2 by tNedd8 (See below). Therefore the co-immunoprecitipation of these constructs may not reflect the protein-protein interaction between Brap2 with tNedd8. Furthermore, Brap2 can dimerize (See below). Therefore, HA-Nedd8 may have immunoprecipitated both HA-Nedd8-conjugated Brap2 and Brap2 constructs that have dimerized. Other deletion mutants (NT R and NT), that did not show doublet band, could still interact with Nedd8. Although the NT domain may be the minimal domain sufficient to interact with Nedd8, the amount of precipitated Brap2 NT was lower than that of Brap2 NT R, suggesting that RING finger domain is important for the binding between Brap2 and Nedd8. Indeed, Brap2 CA mutant that has a single mutation at the cysteine-264 residue in the RING-finger domain (Fig. 2C) could not efficiently co-immunoprecipitate tNedd8 and neddylated proteins (Figs. 2D and 2E), suggesting that the RING finger domain is important for Nedd8 binding.

### Brap2 is neddylated at lysine-432 in vivo

**Figure 3:**
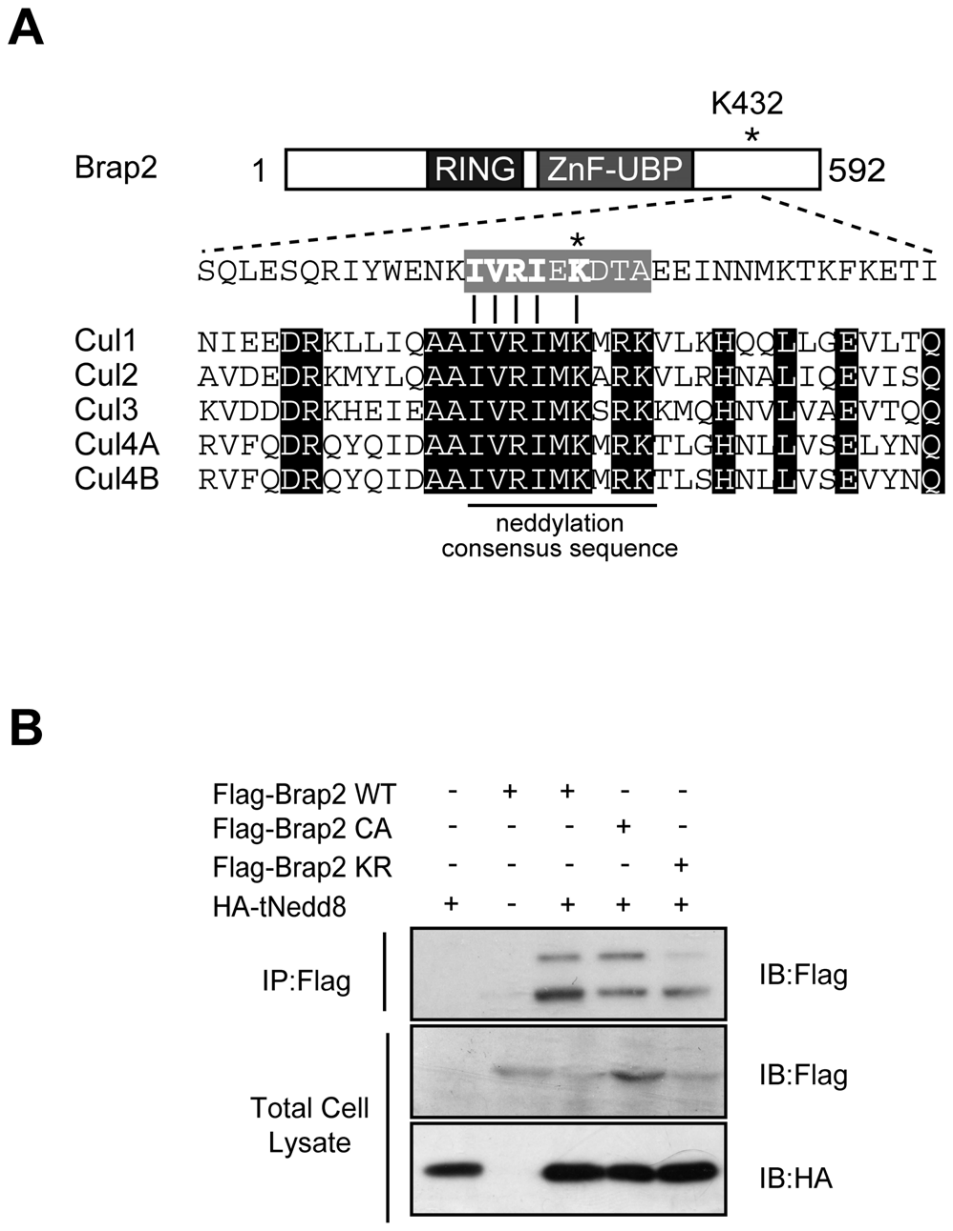
Brap2 is neddylated at lysine-432 *in vivo*. (A) Alignment of putative neddylation site of Brap2 with the consensus neddylation site of cullin family proteins. (**B**) HEK293 cells were transfected with indicated plasmids and were subjected to immunoprecipitation (IP) with Flag antibody and the resulting immunoprecipitant (IP) and total cell lysates were immnoblotted (IB) with indicated antibodies.

In the above experiment, we noticed that doublet bands appeared when Brap2 was immunoprecipitated by tNedd8 (Fig.1C, 2B and 2D). The Brap2 doublet band, that migrates more slowly, appeared when co-expressed with tNedd8. Furthermore, the doublet band appeared when the C-terminal fragments of Brap2, but not the N-terminal fragments were expressed (Fig. 2B). These results suggest that Brap2 can be neddylated and the potential modification site resides in C-terminal region of Brap2. To pursue this idea, we searched for the neddylation site in Brap2. Interestingly, the lysine-432 and its surrounding amino acid sequence were similar to the consensus neddylation sequence conserved in all Cullin family proteins (Fig. 3A) [30]. Moreover, this lysine residue is widely conserved in chordates (Fig. S2). We thus asked whether the lysine-432 residue in Brap2 is capable of being neddylated *in vivo*. Expression of Brap2 WT or CA in the presence of tNedd8 resulted in the appearance of slower migrating bands. However, expression of Brap2 KR, of which lysine-432 is replaced with arginine, led to significantly reduced amount of migrating band (Fig. 3B), indicating that lysine-432 of Brap2 is the potential site of neddylation.

### Brap2 dimerizes without neddylation

**Figure 4:**
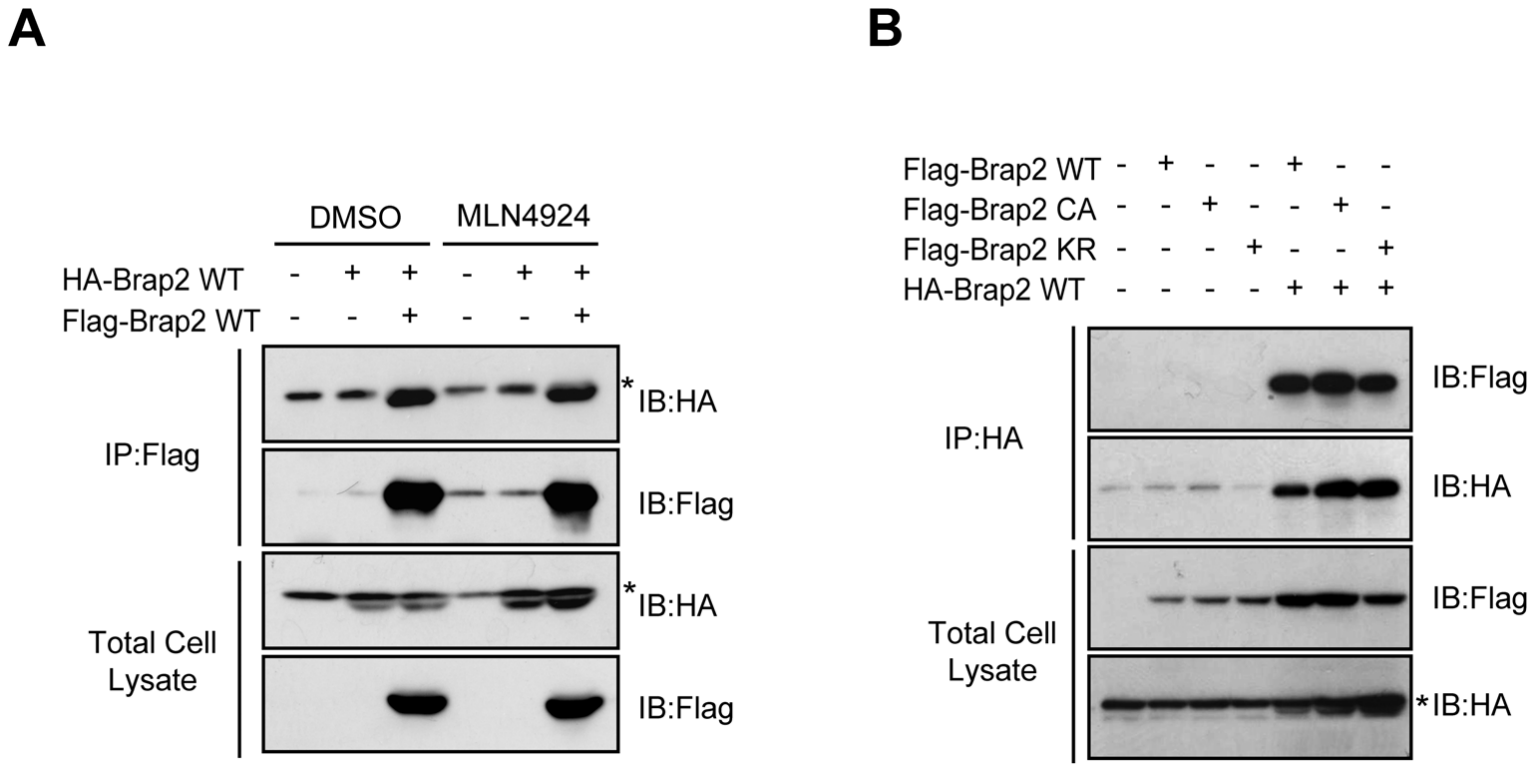
Brap2 dimerizes independent of Nedd8. (A) HEK293 cells were transfected with indicated plasmids and were then subjected to immunoprecipitation with Flag antibody. The total cell lysate and immunoprecipitant (IP) were immunoblotted (IB) using HA and Flag antibodies. Asterisks indicate non-specific bands. (**B**) HEK293 cells were transfected with indicated plasmids and incubated for 6 hours in the presence or absence of 5 µg/ml MLN4924, and were then subjected immunoprecipitation with HA antibody. The total cell lysate and immunoprecipitant (IP) were immunoblotted (IB) using HA and Flag antibodies. Asterisk indicates non-specific bands.

As tNedd8 has immunoprecipitated both modified and unmodified Brap2, we asked whether Brap2 has associated with neddylated Brap2 by forming a dimer or oligomer. To examine this, we transfected HA-Brap2 and Flag-Brap2 into HEK293 cells, incubated the cells in the presence or absence of MLN4924, an inhibitor of Nedd8 E1, and then subjected to co-immunoprecipitation analysis. Flag-Brap2 clearly interacted with HA-Brap2 and treatment with MLN4924 did not block this binding between Flag-Brap2 and HA-Brap2 (Fig. 4A), indicating that the neddylation is not essential for dimer or oligomer formation. To further confirm this, we carried out co-immunoprecipitation assay using a neddylation site mutant of Brap2. Immunoprecipitation of HA-Brap2 WT resulted in co-immunoprecipitation of Flag-Brap2 KR (Fig. 4B). Brap2 CA mutant that does not interact with Nedd8 was also co-immunoprecipitated. Taken together, these data suggest that Brap2 binds to another Brap2, but neddylation is not necessary for this interaction.

### Brap2 associates with Cul1 in a neddylation-independent manner

**Figure 5:**
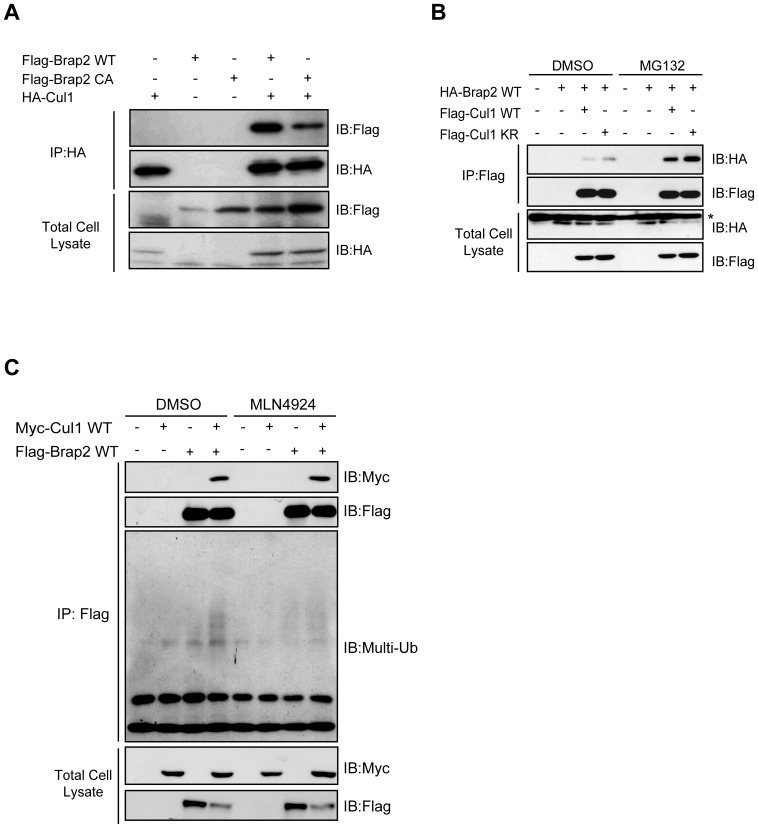
Brap2 associates with Cul1 in a neddylation-independent manner. (A) HEK293 cells were transfected with HA-Cul1 and Flag-Brap2 WT or CA. HA-Cul1 was immunoprecipitated and total cell lysates and immunoprecipitants (IP) were immunoblotted (IB) with Flag and HA antibodies. (**B**) HEK293T cells were transfected with indicated plasmids and incubated for 1 hour in the presence or absence of 20 µM MG132, and subjected to immunoprecipitation (IP) analysis. Asterisk indicates non-specific bands. (**C**) HEK293T cells were transfected with indicated plasmids and incubated for 4 hours in the presence or absence of 1 µM MLN4924, and subjected to immunoprecipitation (IP). The immunoprecipitants (IP) and the total cell lysates were immunoblotted (IB) with indicated antibodies.

It has been reported that Brap2 is implicated in the NF-κB pathway [11], [12]. In addition, Cul1, a component of SCF^β-TrCP^ that plays pivotal roles in NF-κB pathway, is modified by covalent conjugation with Nedd8. Thus, our finding that Brap2 binds to tNedd8 and neddylated proteins led us to ask whether Brap2 can interact with Cul1. To test this hypothesis, we co-expressed HA-Cul1 and Flag-Brap2 WT or CA mutant in HEK293 cells. Immunoprecipitaion of HA-Cul1 could co-immunoprecipitate Flag-Brap2 WT, but Flag-Brap2 CA to a lesser extent (Fig. 5A), suggesting that RING-finger domain is also important, though not essential, for the association with Cul1.

**Figure 6:**
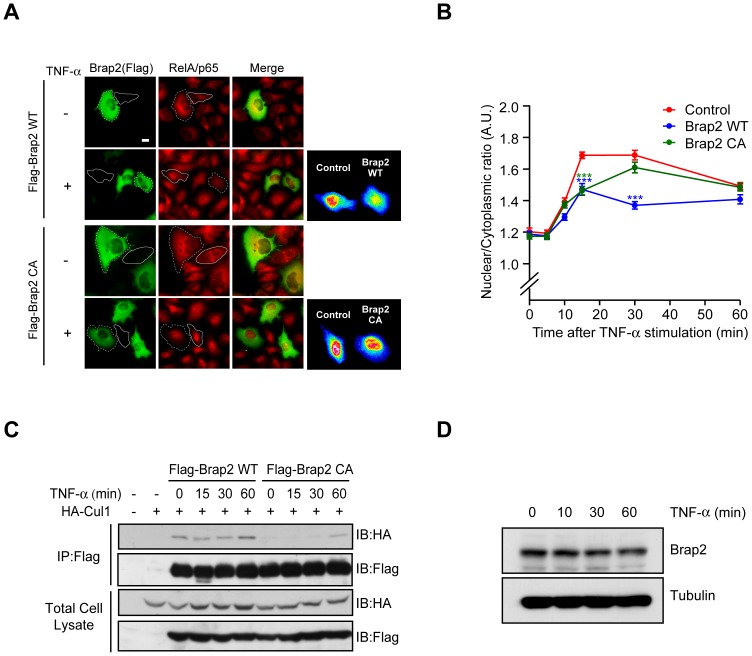
Brap2 attenuates TNF-α-induced NF-κB translocation to nucleus. (A) HeLa cells were transfected with either Flag-Brap2 WT or Flag-Brap2 CA and stimulated with or without 5 ng/ml TNF-α for 30 min, and were subjected to immunocytochemistry using anti-Flag or anti-RelA/p65 antibodies. Cells that express certain amount of Flag-Brap2 are marked by dot lines and control cells are marked by solid line. RelA/p65 translocates in nucleus after the stimulation. The right panels show the same cells using a rainbow color. Bars, 50 µm. (**B**) Ratiometric measurement of RelA/p65 fluorescence observed in cells expressing Flag-Brap2 before and after treatment with 5 ng/ml TNF-α (n = 50; mean ± SEM; *** p<0.0001 by one-way ANOVA). (**C**) HEK293T cells were transfected with HA-Cul1 and Flag-Brap2 WT or CA, and stimulated with or without 5 ng/ml TNF-α for the indicated time, and subjected to immunoprecipitation (IP) with Flag antibody. The total cell lysates and IP were immunoblotted (IB) with indicated antibodies. (**D**) HEK293 cells were stimulated with 5 ng/ml TNF-α for indicated time, and cell lysates were subjected to immunoblot analysis.

We next asked whether neddylation of Cul1 is required for the interaction with Brap2. HEK293T cells were transfected with HA-Brap2 WT and Cul1 WT or a neddylation site mutant of Cul1 in which lysine-720 was replaced with arginine (Cul1 KR) and subjected to immunoprecipitation experiment. Unexpectedly, the amount of immunoprecipitated Flag-Cul1 KR by HA-Brap2 was similar to that of Flag-Cul1 WT, indicating that Brap2 can associate with Cul1 in a neddylation-independent manner (Fig. 5B). To further confirm this effect, we treated cells with MLN4924 for 4 hours and blocked the neddylation cascade *in vivo*. Consistent with our data in Fig. 5B, treatment with MLN4924 did not attenuate the binding between Flag-Brap2 and Myc-Cul1 (Fig. 5C). Interestingly, immunoprecipitants of Flag-Brap2 were slightly ubiquitinated when Myc-Cul1 was overexpressed, and this effect was blocked by treatment with MLN4924. Furthermore the interaction between Brap2 and Cul1 increases in the presence of a proteasome inhibitor MG132 (Fig. 5B), these data imply that SCF ubiquitin ligase promotes ubiquitination of Brap2 for proteasomal degradation (see also Fig. 6D).

### Brap2 attenuates TNF-α-induced NF-κB nuclear translocation

We next examined whether Brap2 regulates TNF-α-induced NF-κB pathway. To test this, we transfected HeLa cells with Brap2, and treated them with 5 ng/ml TNF-α for 30 minutes, and examined the nuclear translocation of NF-κB. We found that expression of Brap2 partially suppresses RelA/p65 translocation to the nucleus in response to TNF-α stimulation. On the other hand, expression of Brap2 CA, which does not efficiently bind to tNedd8 or Cul1, had little effect on RelA/p65 translocation (Fig. 6A). The intensities of RelA/p65 in the nucleus peaked at 15 min and gradually decreased by 60 min in the control cells. Brap2-expressing cells also showed a peak at 15 min but its level was significantly lower compared to control cells (Fig. 6B). Brap2 CA mutant did not inhibit the accumulation of RelA/p65 in the nucleus, but its peak was somehow delayed compared to control cells (Fig. 6B), probably owing to its ability to bind Cul-1 in a Nedd8- and RING-finger independent manner. At 60 min post stimulation, the intensities of nuclear RelA/p65 gradually decreased and their levels were not significantly different between Brap2 WT, CA and control cells. We also tested whether neddylation of Brap2 affects RelA/p65 translocation. Expression of KR mutant could suppress the TNF-α-induced RelA/p65 translocation similar to that of WT (Fig. S3), suggesting that Nedd8 conjugation of Brap2 is not essential for the inhibition of NF-κB translocation. Taken together, these data suggest that Brap2 attenuates NF-κB pathway by regulating the timing of NF-κB translocation.

To further explore the molecular mechanisms by which Brap2 mediates the NF-κB translocation, we examined whether stimulation with TNF-α alters the interaction of Brap2 with Cul1. To examine this, we transfected HEK293T cells with Flag-Brap2 and HA-Cul1, then immunoprecipitated Flag-Brap2 before and after treatment with 5 ng/ml TNF-α. HA-Cul1 associated with Flag-Brap2 WT even in resting cells, and the amount of precipitated HA-Cul1 was not changed, if not slightly increased, after 1 hour of TNF-α stimulation (Fig. 6C). The association with CA mutant was very low at unstimulated condition, but slightly increased at 1 hour of TNF-α stimulation. These results raised the possibility that Cul1 and Brap2 can interact in a RING-finger domain dependent and independent manner during the TNF-α stimulation.

Because Cul1 can induce ubiqitination of Brap2 or its binding target (Fig. 5C), we asked whether TNF-α stimulation promotes degradation of Brap2. Treatment with 5 ng/ml TNF-α slightly decreased endogenous Brap2 protein level after 1 hour of stimulation (Fig. 6D). Taken together, these data suggest Cul1 and Brap2 may have different mode of interaction during the TNF-α stimulation and lead to degradation of Brap2.

### Neddylation of Brap2 is associated with TNF-α-induced NF-κB activity

**Figure 7:**
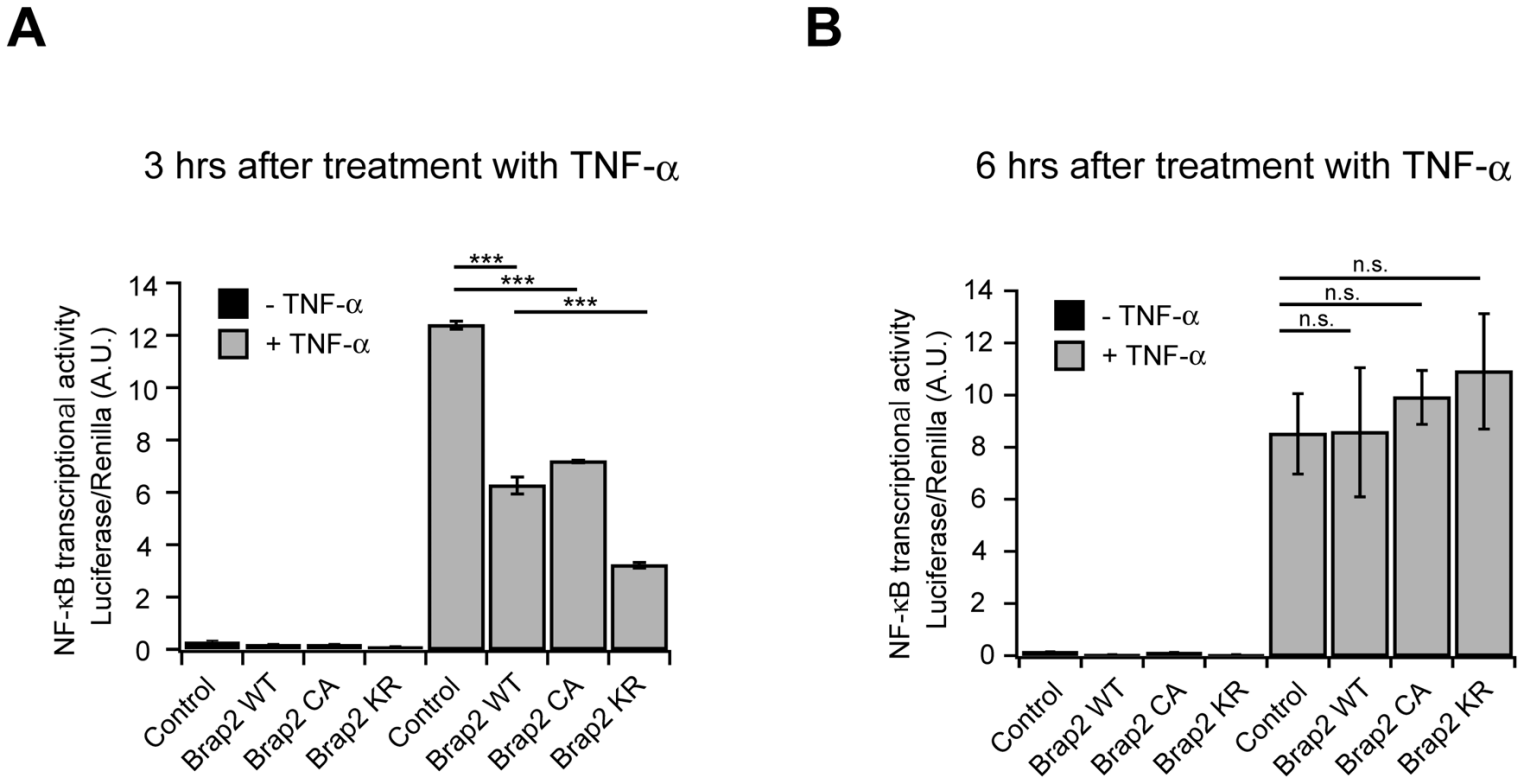
Neddylation of Brap2 is associated with TNF-α-induced NF-κB activity. (A) TNF-α-dependent activation of a NF-κB reporter gene in HEK293 cells expressing indicated plasmids. Cells were stimulated with 5 ng/ml TNF-α for 3 hours. (n = 3; mean ± SEM; *** p<0.0001 by one-way ANOVA). (**B**) TNF-α-dependent activation of a NF-κB reporter gene in HEK293 cells expressing indicated plasmids. Cells were stimulated with 5 ng/ml TNF-α for 6 hours. (n = 3; mean ± SEM; n.s., not significant by one-way ANOVA).

We finally measured the NF-κB transcriptional activity using a luciferase reporter gene assay. Expression of a series of Brap2 constructs suppressed NF-κB transriptional activity after 3 hours of TNF-α stimulation. Interestingly, Brap2 KR was more effective compared to Brap2 WT and CA (Fig. 7A), suggesting that neddylation of Brap2 can alter the conformation or binding affinity with Cullins or its target proteins that modify TNF-α-induced NF-κB activation.

Because the effects of Brap2 mutants on nuclear translocation of NF-κB were significantly different during the time course of TNF-α stimulation, we measured the promoter activities at later time point. The promoter activities in response to TNF-α were not significantly different among each Brap2 mutant expressing cells at later lime point, such as 6 hours of TNF-α stimulation (Fig. 7B), which is in part consistent with the fact that the amount of nuclear NF-κB between Brap2-expressing cells and control cells is similar at 1 hour post TNF-α stimulation (Fig. 6B).

## Discussion

In this study, we identified Brap2 as a Nedd8-binding protein, using yeast two-hybrid screening. Brap2 did not recognize monomeric Nedd8, but associates with smeary bands positive with Nedd8. This suggests that Brap2 binds to poly- or multiple-neddylated proteins. Recent proteomic analysis has revealed that Nedd8 covalently conjugates to another Nedd8 in stable cell lines expressing GST-Nedd8 [31]. Also poly-neddylation was observed using an *in vitro* reconstitution assay [32], and in the cells expressing Nedd8 [33]–[34]. Thus, it is conceivable that Nedd8 can form polymeric chains in a context-dependent manner, and Brap2 may have preference to recognize polymeric Nedd8 chains.

In an attempt to identify the Nedd8-binding domain of Brap2, we found that Brap2 binds to Nedd8 via non-covalent and covalent manner. Brap2 possesses the consensus neddylation sequence conserved in Cullin family proteins and it appeared that Nedd8 covalently conjugates at this site, although further verification by mass-spectrometry is required. As the potential neddylation site resides in CT domain, all Brap2 constructs that contain this domain could be immunoprecipitated by the covalent conjugation with HA-tNedd8. It was reported that BRIZ1 and BRIZ2, Brap2 homologs in *Arabidopsis* can heterodimerize through CT domain [35]. The CT domain is required and sufficient for the dimer formation of BRIZ1 and BRIZ2. As BRIZ1 and BRIZ2 do not have consensus neddylation site, it is conceivable that neddylation is not important for dimerization. Indeed, KR mutation or the addition of MLN4924 did not block the dimer formation. Alternately, neddylation at the CT domain may inhibit the dimer formation of CT domain. Although this possibility remains, it appears that Nedd8-conjugated Brap2 can dimerize with unconjugated form. When the ratio of Nedd8 conjugated and unconjugated forms were compared among mutants that contain CT domain, equivalent amount of conjugated and unconjugated forms were immunoprecipitated despite of the apparently low amount of Nedd8-conjugated form in the total cell lysate. This suggests that conjugated form was efficiently immunoprecipitated by Nedd8, and the unconjugated form of CT domain might be co-precipitated indirectly by the heterodimerization of CT domain. Therefore, CT domain may not be the domain that binds Nedd8 non-covalently.

Besides, Nedd8 could coimmunoprecipitate Brap2 mutants that do not contain CT domain. Since these mutants (NT and NT R) are not subjects of Nedd8 conjugation, the association may reflect the protein-protein interaction. Although we do not exclude the possibility that NT domain is the minimal domain sufficient for non-covalent binding with Nedd8, mutant that contains the RING finger domain was more efficient for the binding. Indeed, single mutation at the RING finger domain reduced the Nedd8-binding although it contained NT domain, supporting our notion that RING-finger domain is important for Nedd8 interaction. Further analysis to indentify its direct interaction is needed in future.

Brap2 can associate with Cul1, a well-known target protein conjugated with Nedd8. Despite the binding of Brap2 and Cul1 can be mediated by Nedd8, it was independent of neddylation. Cul1 KR mutant could bind Brap2 and MLN4924 did not block the interaction of Cul1 and Brap2. Furthermore, Brap2 CA, which cannot bind to Cul1 or Nedd8 at unstimulated condition, could interact with Cul1 post-TNF-α stimulation. This raises the possibility that Brap2 and Cul1 have different mode of interaction that do not involve the RING finger domain and Nedd8.

Although Nedd8 is not required for the association of Brap2 with Cul1, it is tempting to investigate how neddylation affects the function of Brap2 and Cul1 in the complex. One possibility is that Cul1 neddylation promotes the ubiquitination of Brap2, which targets it for degradation. Indeed we could observe Brap2 ubiquitination in the presence of Cul1 and its slight degradation upon TNF-α stimulation. The other possibility is that, ubiquitin-positive smear bands associated with Brap2 reflect the activation of presumptive E3 ligase activity of Brap2 in the presence of Nedd8 modification. Further analysis is required to clarify the functional interaction of Brap2 and Cul1.

**Figure 8:**
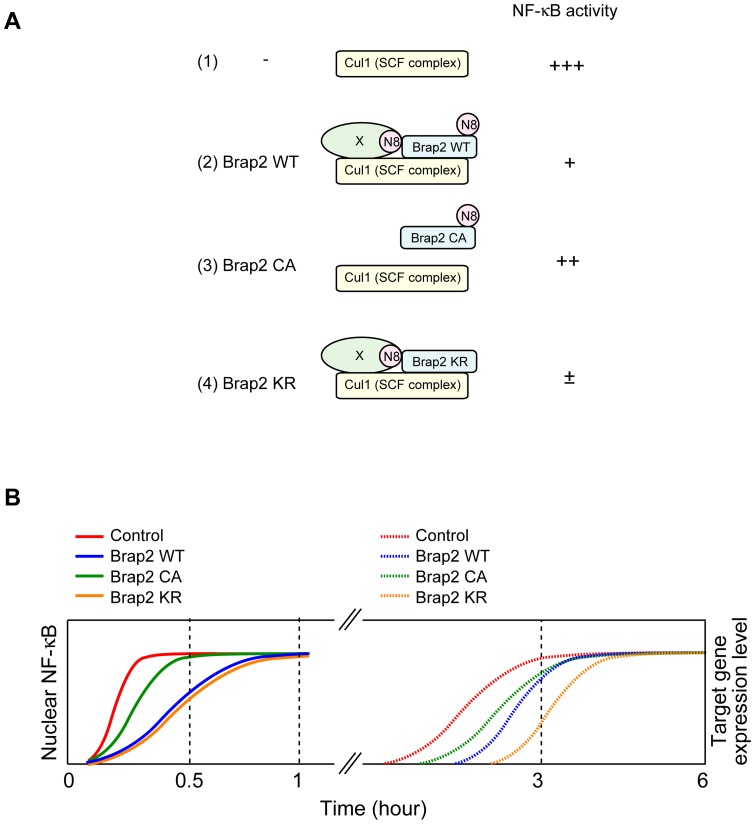
Model of Brap2 function. (A) Model of Brap2 interaction with Cul1 and its effect on NF-κB signaling pathway. (**B**) Time course of NF-κB translocation and target gene expression by each Brap2 constructs.

The expression of Brap2 delays TNF-α-induced NF-κB translocation to the nucleus and the mutant Brap2 that lacks a neddylation site suppressed NF-κB activity more efficiently than the wild type (Fig. 7A and 8A). The Brap2 CA mutant that cannot bind to Cul1, did not exert such inhibitory effects. These results suggest that the functions of Cul1 are disturbed by overexpression of Brap2. Furthermore, neddylation of Brap2 may have negative impact on the function of Brap2. However, contrary to our data, it was reported that knockdown of Brap2 suppresses NF-κB activation following LPS stimulation [12]. This discrepancy may have been arisen from the complex interaction between Brap2 and Cul1. As Brap2 and Cul1 appears to have two different mode of interaction, it is possible that Brap2 knockdown and overexpression have affected different states. For instance, the initial state can be affected by the knockdown but not overexpression, while the latter state by the both. If Brap2 cooperates with Cul1 at initial state but antagonizes at the later, both the knockdown and overexpression can lead to suppression of NF-κB activation. Furthermore, the overexpression of Brap2 may not reflect the gain-of-function phenotype. The other possibility is that we only analyzed NF-κB translocation to the nucleus and promoter activity in Brap2-expressing cells, and did not investigate other NF-κB signaling process. Thus, it is possible that Brap2 also targets not only SCF^β-TrCP^ but also another protein that is implicated in the NF-κB pathway.

Recent studies have reported that the oscillation frequency of NF-κB activation regulates different kinds of gene expression. Short exposure to TNF-α produces a short pulse of NF-κB activation and expression of a subset of target genes. On the other hand, sustained exposure to TNF-α leads to the nuclear-cytoplasmic shuttling of NF-κB, resulting in expression of another set of genes [36], [37]. Because sustained exposure to TNF-α increased the binding between Brap2 and Cul1, Brap2 may play an important role in NF-κB translocation efficiency. In this regard, it is possible that Brap2 alters the timing of translocation and controls the oscillation frequency of NF-κB activation and target gene expression, in response to both acute and sustained inflammatory stimulation. Indeed, the expression level of luciferase in Brap2-expressing cells was lower than that in the control cells after 3 hours of TNF-α stimulation, but its levels were not significantly different after 6 hours of stimulation, which was in accordance with the level of NF-κB in the nucleus at later time point (see Fig.6A and 8B).

Taken together, our result demonstrates that Brap2 is a novel modulator that mediates the NF-κB pathway under inflammatory conditions, through a mechanism that involves Cul-1 and/or other potential neddylated proteins. Given that Brap2 associates with many human disorders related to dysregulated inflammatory responses, further analysis on Brap2-Cul1 functional interaction would provide a novel clue to understand the pathology of those diseases and development of therapeutics [11]–[13], [38].

## Materials and Methods

### Antibodies and reagents

Antibodies to Flag (M2, SIGMA), HA (A190-108A, Bethyl Lab., Inc), HA (Y-11, Santa Cruz), Ubiquitin (FK2, MBL), Brap2 (A302-682A, Bethyl Lab., Inc) were used for immunoblot analyses, HA (Y-11, Santa Cruz) and RelA/p65 (C22B4, Cell Signaling) for immunocytochemistry, and Anti-Flag M2-agarose and anti-HA-agarose beads (SIGMA) for immunoprecipitation. MG132 (Peptide Institute), MLN4924 (Toronto Research Chemicals), and TNF-α (R&D Systems) were purchased.

### Cell culture and transfection

HEK293, HEK293T and HeLa cells were cultured in Dulbecco”s modified Minimal Essential Medium (WAKO) containing 5–10% fetal bovine serum, penicillin (100 units/ml) and streptomycin (100 µg/ml). Cells were transfected with plasmids using Lipofectamine 2000 (Invitrogen) and FuGENE (Roche) according to the manufacturer”s instructions.

### Plasmid construction

Nedd8 constructs were cloned into the *Eco*RI and *Not*I sites of pBG4D-2 and pcDNA3.1-HA. Brap2 was amplified by Marathon cDNA Amplification Kit (Clontech) from human cDNA library and subcloned into the *Eco*RI and *Not*I sites of pcDNA3.1-Flag. Site directed mutagenesis was performed using Quick Change (Stratagene) to generate the Brap2 mutant. The Cul1 constructs were described previously [32].

### Yeast two-hybrid screening

Plasmids were transformed into the *Saccharomyces cerevisiae* strain PJ69-4A. The pBG4D-2-tNedd8 was used as bait and screened using a mouse embryonic (E11) cDNA library. The two-hybrid screening was performed according to the manufacturer”s instructions. Interactions were confirmed by co-transformation with the isolated prey vectors and the tNedd8 bait vector.

### Immunoblot analysis

Cells were lysed in extraction buffer (20 mM Tris-HCl (pH 7.5), 150 mM NaCl, 1 mM EDTA, 0.5% NP-40) and centrifuged at 15,000 rpm for 20 minutes. The cleared lysates were separated by SDS-PAGE, transferred to PVDF membrane, probed with primary antibodies, and detected with HRP-conjugated secondary antibodies and chemiluminescence reagent (Amersham ECL Plus Western Blotting Detection Reagents, GE Healthcare).

### Immunoprecipitation

The cell lysates (see immunoblot analysis) was rotated with anti-Flag M2 agarose or anti-HA agarose beads for 2 hours at 4°C. The immunoprecipitants were washed and subjected to immunoblot analyses with antibodies to Myc, Flag and HA.

### Immunocytochemistry

HeLa cells plated on 12 mm coverslips and grown in 12-well plates were fixed with 4% paraformaldehyde in phosphate buffered saline (PBS) for 10 minutes at room temperature. The coverslips were washed in PBS, blocked with 5% bovine serum albumin (BSA) in PBS with 0.4% Triton X-100, then incubated with the indicated primary antibodies for one hour at room temperature or overnight at 4°C. Following PBS wash, samples were incubated with secondary antibodies (Alexa Fluor 488 anti-mouse IgG (1∶500), and Alexa Fluor 594 anti-rabbit IgG (1∶500)) for 30 minutes at room temperature in blocking solution. Cells were imaged using a fluorescence microscope (Keyence, BIOREVO BZ-9000). Fluorescence images were analyzed using Image J.

### Luciferase assay

HEK293 cells were transfected with the indicated expression vectors as well as with the NF-κB reporter plasmid that encodes firefly luciferase, and a control plasmid that encodes renilla luciferase. Cell lysates were subsequently assayed for both firefly and renilla luciferase activities with Dual-Luciferase Reporter Assay System (Promega), and the former activity was normalized on the basis of the latter.

### Statistical analysis

Statistical significance was analyzed by one-way analysis of variance (ANOVA) with Tukey”s multiple comparison test using Prism software (GraphPad Software, Inc.).

## Supporting Information

Figure S1 **Nedd8 forms smear bands.** HEK293 cells were transfected with either HA-Nedd8 or HA-Ub. Cell lysates were subjected to immunoprecipitation (IP) with HA antibody. The immunoprecipitants (IP) were subjected to immunoblot (IB) analyses with antibody to HA.
(TIF)

Figure S2 **Sequence alignment of Brap2.** Conserved sequences are highlighted in black. Asterisk indicates predicted neddylation sites on Brap2.
(TIF)

Figure S3 **Brap2 KR suppresses RelA/p65 translocation similar to Brap2 WT.** HeLa cells were transfected with either Flag-Brap2 WT or Flag-Brap2 KR and stimulated with or without 5 ng/ml TNF-α for 30 min, and were subjected to immunocytochemistry using anti-Flag or anti-RelA/p65 antibodies. Bars, 10 µm.
(TIF)
