# Supplementary figures and images for: Brap2 Regulates Temporal Control of NF-κB Localization Mediated by Inflammatory Response
Source: PLoS One. 2013 Mar 15;8(3):e58911. doi: 10.1371/journal.pone.0058911 (PMC3598860; doi:10.1371/journal.pone.0058911)

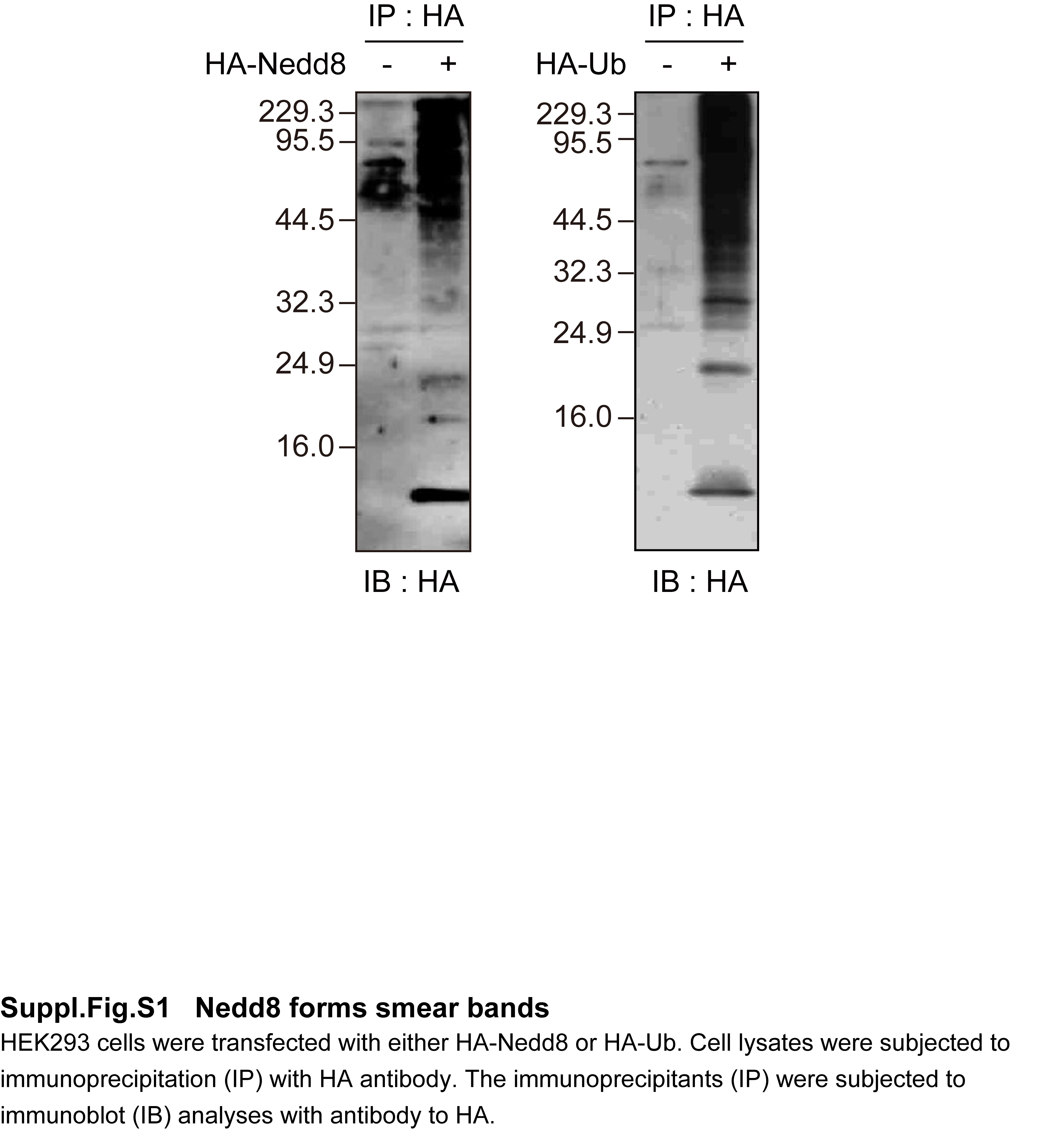

Supplement: Figure S1 — Nedd8 forms smear bands. HEK293 cells were transfected with either HA-Nedd8 or HA-Ub. Cell lysates were subjected to immunoprecipitation (IP) with HA antibody. The immunoprecipitants (IP) were subjected to immunoblot (IB) analyses with antibody to HA. (TIF) [file pone.0058911.s001.tif]

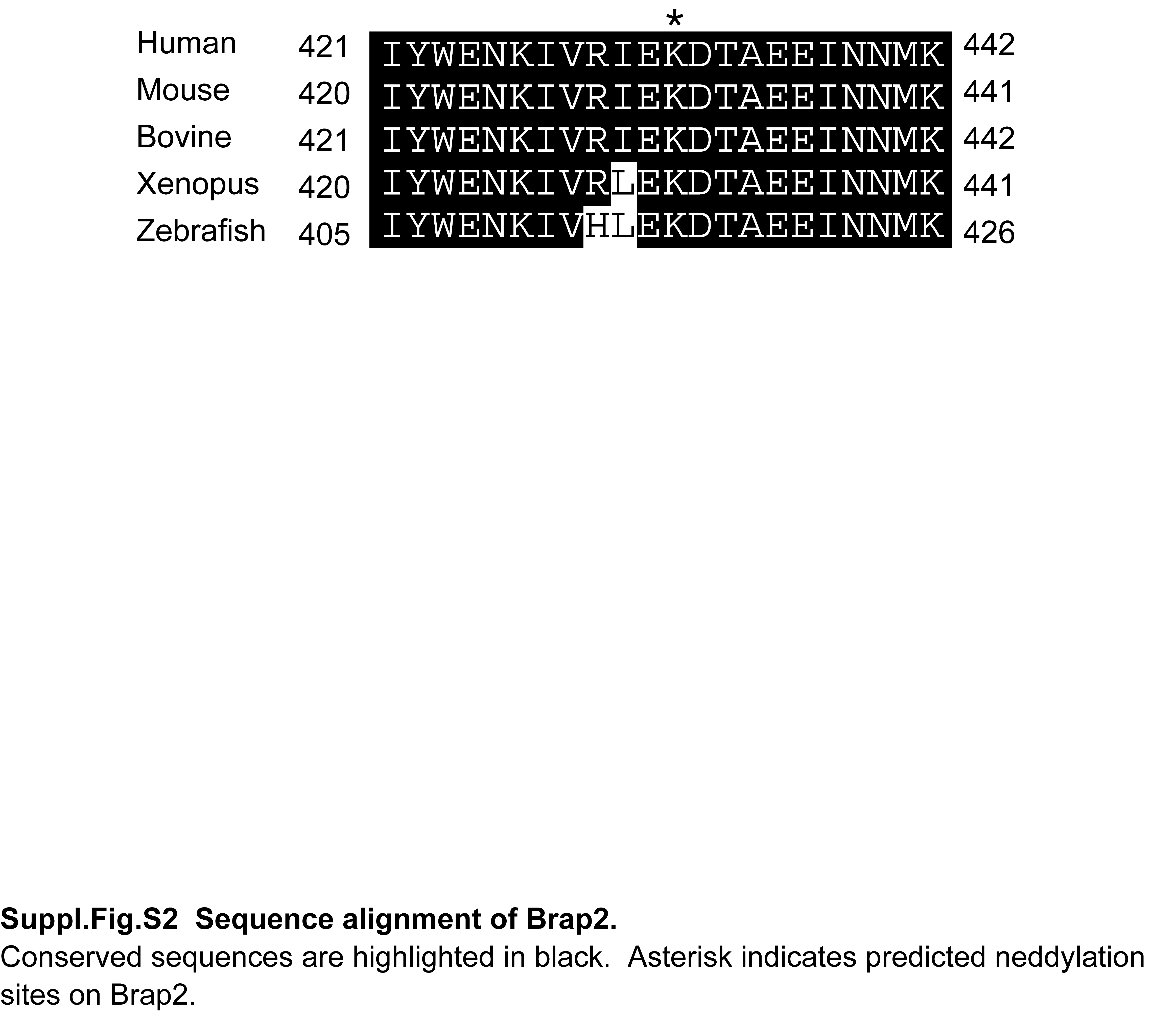

Supplement: Figure S2 — Sequence alignment of Brap2. Conserved sequences are highlighted in black. Asterisk indicates predicted neddylation sites on Brap2. (TIF) [file pone.0058911.s002.tif]

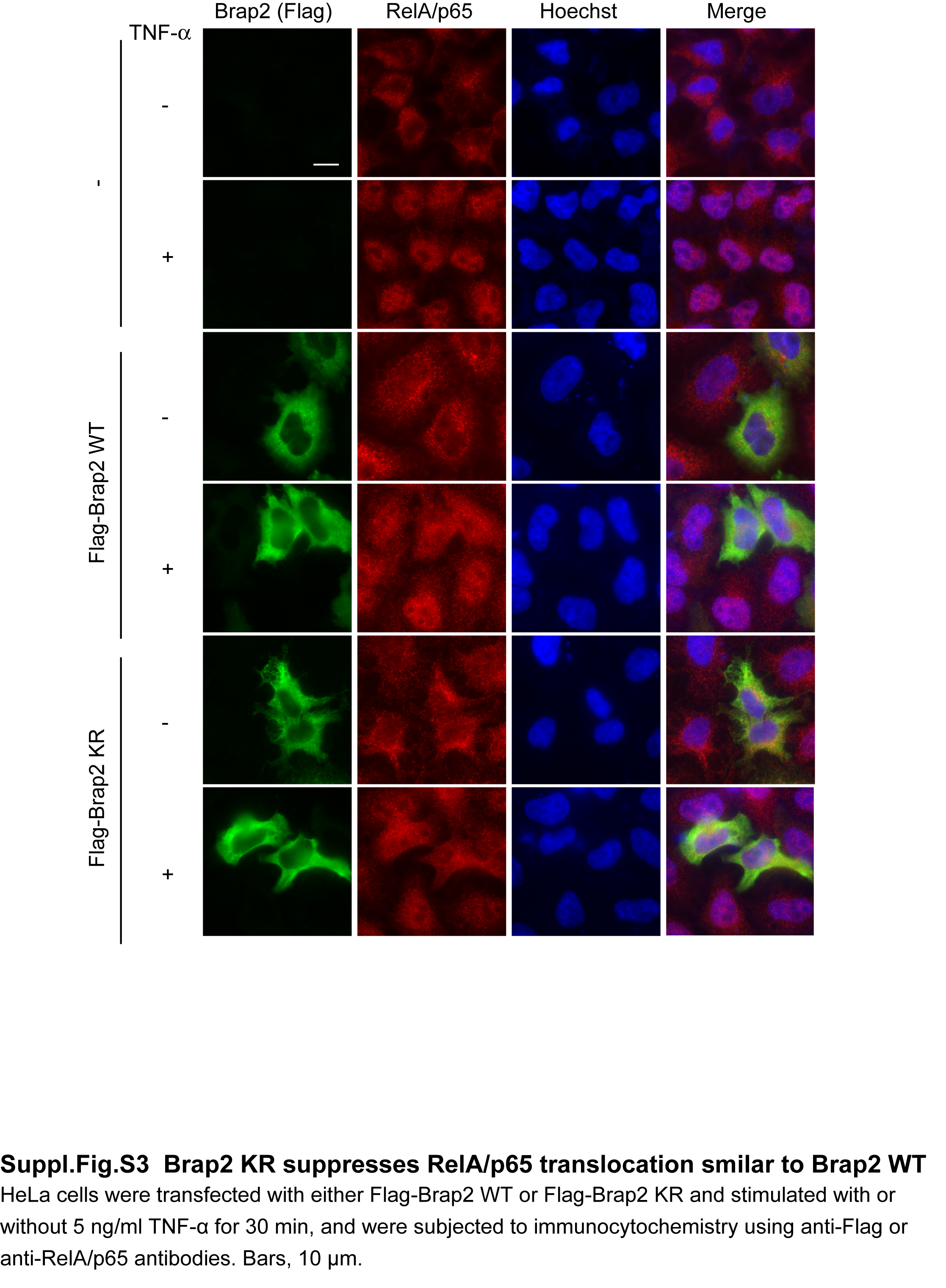

Supplement: Figure S3 — Brap2 KR suppresses RelA/p65 translocation similar to Brap2 WT. HeLa cells were transfected with either Flag-Brap2 WT or Flag-Brap2 KR and stimulated with or without 5 ng/ml TNF-α for 30 min, and were subjected to immunocytochemistry using anti-Flag or anti-RelA/p65 antibodies. Bars, 10 µm. (TIF) [file pone.0058911.s003.tif]
